# DTOFW: a lightweight tri-branch time-frequency fusion network for video-based recognition of diarrhea-related calf behavior

**DOI:** 10.3389/fvets.2026.1859339

**Published:** 2026-07-31

**Authors:** Wangli Hao, Qingqing Li, Lingling Li, Yakui Xue, Meng Han, Yanhong Liu, FuZhong Li

**Affiliations:** 1 Faculty of Software Technologies, Shanxi Agricultural University, Jinzhong, Shanxi, China; 2 School of Information Science and Engineering, Shanxi Agricultural University, Jinzhong, Shanxi, China

**Keywords:** calf diarrhea recognition, DINOv2, lightweight network, neural ODE, time-frequency fusion

## Abstract

Accurate recognition of diarrhea-related behaviors in calves is crucial for automated health monitoring in smart farming. However, existing methods face challenges including insufficient temporal continuity and limited capability in capturing periodic or abrupt behavioral changes. To address these issues, this study proposes a lightweight tri-branch time-frequency fusion network named self-distillation with no labels version 2 (DINOv2)–Transformer ODE (ODE)–Fourier-Wavelet (DTOFW) for calf diarrhea behavior recognition. The network employs DINOv2 as a frozen backbone for high-level semantic feature extraction and integrates three complementary branches for comprehensive behavioral modeling. The Transformer-ODE branch models continuous temporal dynamics to compensate for information gaps caused by discrete sampling. The Fourier attention branch adaptively emphasizes global periodic patterns in the frequency domain. The Wavelet attention branch captures local transient and non-stationary abnormalities associated with diarrhea. Experimental results on the Jinnan calf behavior dataset show that DTOFW achieves 95.32% recognition accuracy with 1.33 million trainable parameters in the tri-branch fusion head. Compared with the tested baseline models, DTOFW improves accuracy by up to 8.71 percentage points while maintaining a low number of trainable parameters in the fusion head. The proposed model provides an effective and efficient solution for fine-grained abnormal behavior recognition in intelligent livestock farming.

## Highlights

- We propose a lightweight tri-branch time-frequency fusion network, self-distillation with no labels version 2 (DINOv2)-Transformer ODE (ODE)-Fourier-Wavelet (DTOFW), for calf diarrhea behavior recognition. It unifies continuous temporal modeling, global frequency perception, and local time-frequency detail extraction into a compact framework, achieving an excellent trade-off between accuracy and computational efficiency.
- We first introduce a Transformer-ODE continuous dynamic modeling module. This module integrates neural ODE with a lightweight Transformer to compensate for temporal information gaps caused by discrete sampling and reliably capture long-range evolutionary dependencies in behavioral sequences.
- We first develop a collaborative time-frequency enhancement mechanism consisting of Fourier attention and Wavelet attention. The former strengthens global periodic patterns, whereas the latter enhances local transient abnormal features, thus significantly improving the discriminative capacity for subtle diarrhea-related behaviors.
- This research first validates a two-stage training paradigm that combines a frozen DINOv2 backbone with a lightweight three-branch head in calf behavior recognition. Achieving 95.32% classification accuracy with only 1.33M parameters, this paradigm supports efficient edge deployment in real farming scenarios.

## 1 Introduction

Calf health strongly affects farming efficiency and animal welfare. Accurate behavior recognition enables early detection of diseases like diarrhea, allowing timely adjustment of feeding management and treatment plans, preventing growth delay and death (1). Therefore, calf behavior recognition serves as a key technology for intelligent health monitoring and an important measure to support sustainable calf farming. Calf diarrhea is a common health problem in early-life calf rearing and may be accompanied by observable changes in activity, lying patterns, and daily behavior patterns (2). These behavioral changes provide useful visual cues for non-contact health monitoring in calf production. Therefore, video-based recognition of diarrhea-related calf behavior can provide auxiliary information for farm health monitoring, although it should not be considered a replacement for veterinary clinical diagnosis.

Traditional calf behavior recognition mainly relies on wearable sensors. Accelerometer-based techniques are widely applied in livestock activity monitoring and enable quantitative capture and analysis of behaviors (3, 4). Machine learning methods based on IMU are also applied to daily behavior pattern recognition in dairy cows, promoting early development of behavior monitoring (5). Wearable sensors, equipped with accelerometers and gyroscopes, can capture animal activity states with certain accuracy and support quantitative monitoring and analysis. Some studies combine wearable nose rings with machine learning classifiers to achieve interpretable monitoring of dairy cow behavior. However, these devices present several inherent limitations. Hardware purchase and maintenance costs remain high, wearing may induce stress in calves, and battery life and data transmission efficiency are limited, making long-term monitoring in large-scale farms difficult (6, 7). More importantly, discrete sampling fails to capture subtle behavioral changes at the early stage of diarrhea, limiting fine-grained abnormal behavior recognition (8). With the rapid development of smart livestock farming, more researchers explore advanced techniques based on deep learning to replace traditional wearable approaches. These methods greatly improve recognition accuracy and efficiency and provide strong technical support for intelligent health monitoring in large-scale farms (9, 10).

In recent years, deep learning has driven animal behavior recognition into a new stage and become a core approach to overcome limitations of traditional methods. Convolutional neural networks, visual Transformers, and their variants are widely applied in this field, enhancing temporal feature modeling and supporting edge deployment for large-scale farms (11). In feature extraction and model optimization, researchers explore multiple directions. Fourier transform can characterize periodic patterns in behavior sequences and supports quantitative analysis, showing good performance in wildlife behavior recognition (12). Deep wavelet transform and multifractal analysis capture nonlinear dynamic features and detect subtle behavior changes, supporting fine-grained recognition (13). Wavelet Transformer studies provide new ideas for combining frequency-domain and temporal features, and efficient designs support lightweight model development (14, 15). The integration of micro-motion feature extraction with attention mechanisms further improves fine-grained recognition accuracy and becomes an important research direction (16–18). Multi-stage recognition strategies show good performance in animals like sheep and offer reference for fine-grained tasks like calf diarrhea detection (19). In addition, recent vision algorithms like YOLOv8 achieve strong performance in detailed feeding behavior analysis of dairy cows (20). Low-power edge deployment also support practical deployment (21). Improved architectures based on SlowFast networks enable behavior recognition across different cattle breeds, providing cross-breed reference for calf behavior analysis (22).

However, current deep learning methods still face two key challenges in fine-grained abnormal behavior recognition for calf diarrhea. Discrete sampling fails to capture continuous dynamics and long-range dependencies in calf behavior (23). Single-domain frequency analysis cannot balance global periodic patterns and local transient changes.

To address these issues, this study proposes a calf diarrhea behavior recognition framework based on time–frequency fusion and continuous dynamic modeling. Specifically, a temporal continuous modeling module, a Fourier global frequency module, and a wavelet local frequency module are constructed in sequence. During feature learning, the three modules jointly extract continuous dynamic features, global periodic features, and local transient features, enabling deep complementarity and efficient fusion of time–frequency information.

The main contributions of this study are summarized in four aspects.

- This paper first proposes a lightweight tri-branch time-frequency fusion network, DTOFW, for calf diarrhea behavior recognition. It unifies continuous temporal modeling, global frequency perception, and local time-frequency detail extraction into a compact framework, achieving an excellent trade-off between accuracy and computational efficiency.
- We first introduce a Transformer-ODE continuous dynamic modeling module. This module integrates neural ODE with a lightweight Transformer to compensate for temporal information gaps caused by discrete sampling and reliably capture long-range evolutionary dependencies in behavioral sequences.
- This work first develops a collaborative time-frequency enhancement mechanism consisting of Fourier attention and Wavelet attention. The former strengthens global periodic patterns, whereas the latter enhances local transient abnormal features, thus significantly improving the discriminative capacity for subtle diarrhea-related behaviors.
- This study validates a two-stage training paradigm that combines a frozen DINOv2 backbone with a lightweight tri-branch fusion head for calf behavior recognition. The model achieves 95.32% classification accuracy with 1.33M trainable parameters in the fusion head, indicating a favorable balance between recognition performance and training efficiency.

## 2 Dataset and implementation

### 2.1 Dataset

**Figure 1:**
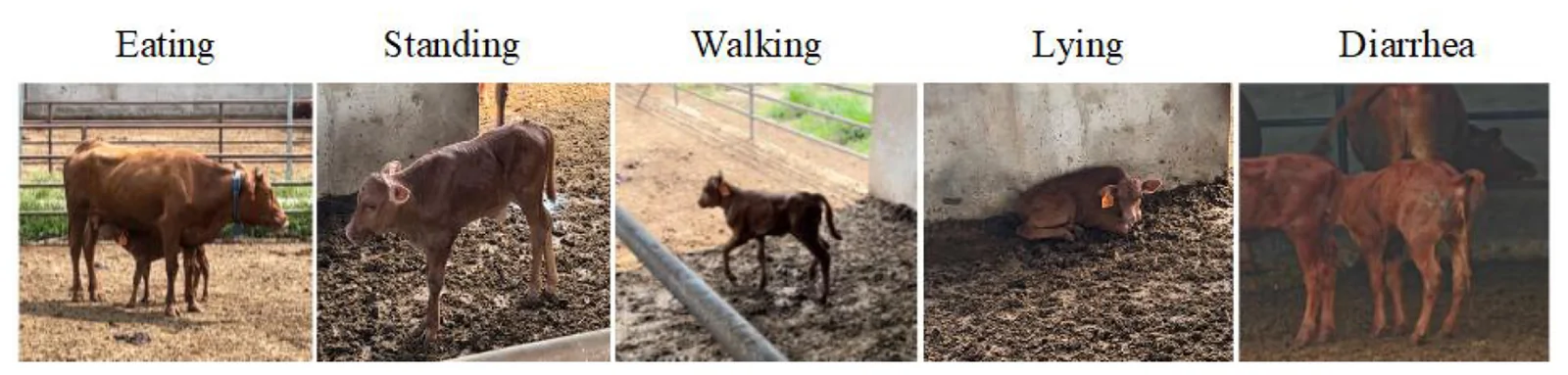
Visual examples of five behavior categories in the Jinnan calf dataset.

The Jinnan calf diarrhea behavior dataset was collected at the National Jinnan Cattle Genetic Resource Conservation Center in Yuncheng, Shanxi Province. The dataset contains recordings from 45 individual Jinnan calves aged 0 to 6 months. Figure 1 shows five representative behavior categories in the dataset: diarrhea-related behavior, eating, lying, standing, and walking. A total of 2,359 video clips were sampled at 25 frames per second and clipped to 5–10 s per sample. All videos were captured using Hikvision DS-2DE3Q120MY-T/GLSE RGB cameras with a resolution of 1, 920 × 1, 080 pixels at 25 frames per second. A total of 12 cameras were installed approximately 2.5 m above the ground at the four corners of each barn, with viewing directions of 45°, 135°, −45°, and −135°, enabling complete coverage of the calf activity area. Researchers manually reviewed each complete video clip and assigned behavior labels according to predefined annotation guidelines. The diarrhea-related behavior category was annotated at the video-clip level by reviewing complete calf video sequences, and mainly reflects visually observable defecation-related movements and associated body-centered behavioral cues, rather than isolated fecal appearance or cropped hindquarter-contamination features. All videos were captured under routine farm housing conditions with diverse illumination and time periods to ensure authenticity and diversity. The dataset is split into training and testing sets at a ratio of 8:2, as shown in Table 1. To ensure a rigorous evaluation, video clips from the same animal strictly do not appear in both the training and test sets. Each video is uniformly sampled to 16 frames, and data augmentation including random cropping and flipping is applied to the training set to improve generalization.

**Table 1:** Dataset statistics of behavior categories.

Behavior category	Diarrhea-related	Eating	Lying	Standing	Walking
Number	271	385	509	634	560
Training	216	307	406	507	448
Test	55	78	103	127	112

The 271 diarrhea-related samples reported in Table 1 refer to video clips assigned to the diarrhea-related behavior category, rather than clinically confirmed diarrhea episodes.

### 2.2 Implementation

All experiments were conducted on a workstation equipped with an Intel Xeon Gold 6248 CPU (Intel Corporation, Santa Clara, CA, USA) and an NVIDIA GeForce RTX 3090 GPU with 24 GB of memory (NVIDIA Corporation, Santa Clara, CA, USA), running Ubuntu 18.04. The network was implemented using PyTorch 1.12.0 (Meta Platforms, Inc., Menlo Park, CA, USA) with CUDA 11.6 (NVIDIA Corporation, Santa Clara, CA, USA). The batch size was set to 32, and the model was trained for 100 epochs using an adaptive learning rate optimizer.

## 3 Methods

**Figure 2:**
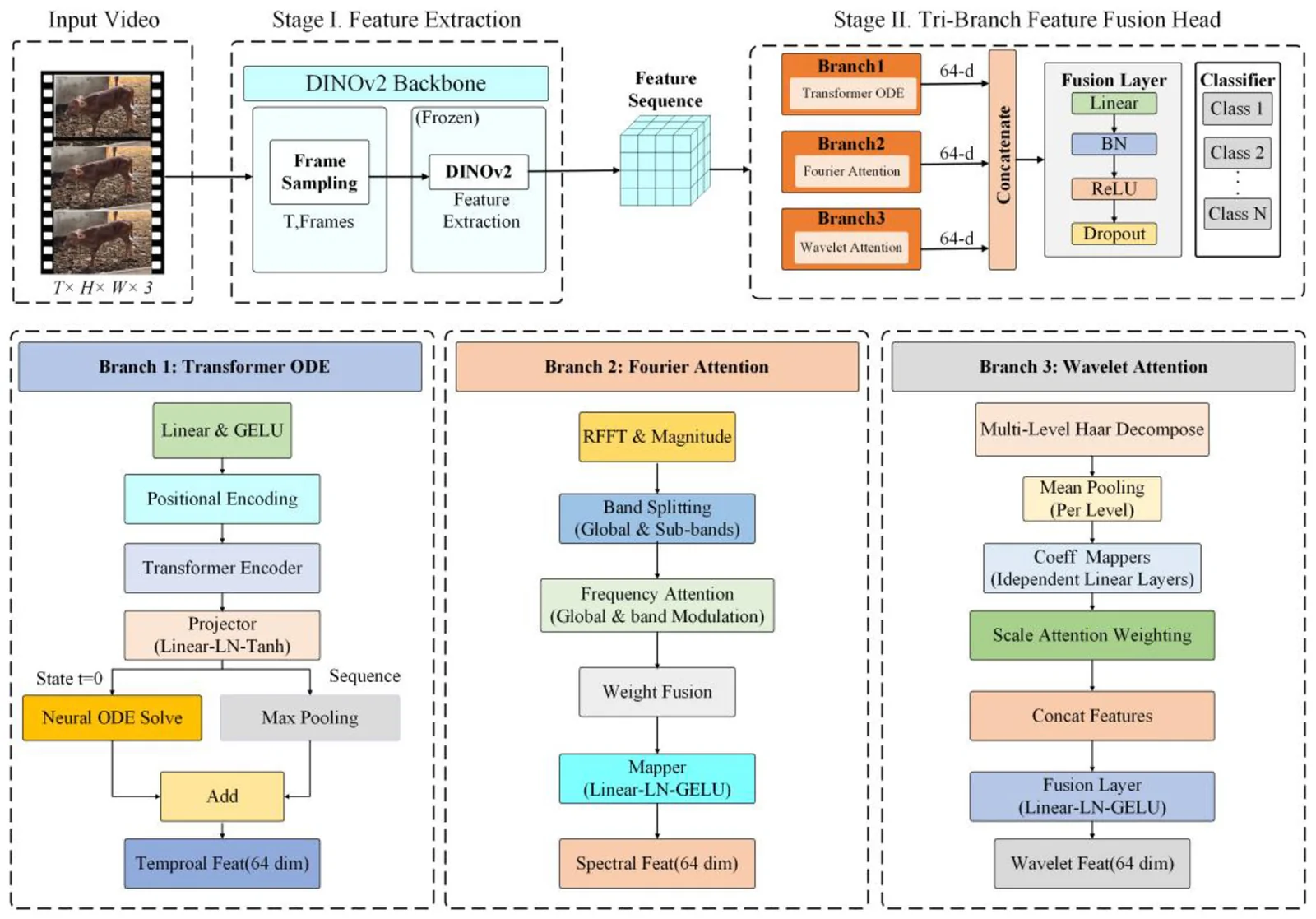
Overall architecture of the DTOFW network.

To address the limits of current behavior recognition methods in discrete temporal modeling, a lightweight three-branch time-frequency fusion network, DTOFW, is proposed. It also addresses the insufficient use of frequency-domain information and the weak adaptation to edge deployment. The network is developed for diarrhea behavior recognition in southern Shanxi calves. A two-stage training strategy was adopted. In the first stage, the DINOv2 backbone remains frozen, and offline feature extraction and caching are performed on video frames. In the second stage, only the lightweight three-branch fusion head is trained. Continuous temporal modeling, global frequency-domain modeling, and local time-frequency detail modeling can therefore be completed in a low-dimensional space. This design not only reduces the risk of overfitting in small-sample scenarios, but also greatly improves training efficiency and compatibility with edge deployment. The overall architecture is shown in Figure 2.

### 3.1 DTOFW network architecture

#### 3.1.1 Visual backbone: frozen DINOv2

To obtain discriminative frame-level semantic representations with limited trainable parameters, DINOv2 pre-trained via self-supervised learning is employed as the visual backbone with parameters frozen during training. Given an input video uniformly sampled into *T* frames, the *t*-th frame is denoted as *I*_*t*_. Each frame is independently fed into the DINOv2 encoder to generate frame-level features. The frame-level semantic features are extracted using Equation 1.


$$\begin{array}{c} f_{t}=\text{DINOv2}\left(I_{t}\right) \end{array}$$ (1)

where *f*_*t*_ denotes the frame-level feature vector, and the single-frame feature dimension *D* = 768. All frame features are stacked in temporal order to construct the video-level temporal feature matrix. The video-level temporal feature matrix is constructed using Equation 2.


$$\begin{array}{c} X=\left[f_{1},f_{2},\ldots ,f_{T}\right] \end{array}$$ (2)

where *X*∈ℝ^*T*×*D*^ represents the video-level feature sequence. For batch processing, the input tensor is extended to *X*∈ℝ^*B*×*T*×*D*^, where *B* denotes the batch size, *T* denotes the sequence length, and *D* denotes the feature dimension of a single frame. The frozen DINOv2 stabilizes high-level semantic feature extraction, reduces training complexity, and provides unified input for subsequent three-branch modeling. While the DTOFW head contains only 1.33M trainable parameters, the overall system relies on a frozen DINOv2 backbone, which contributes additional non-trainable parameters during inference. The term “lightweight” in this work specifically refers to the trainable fusion head, as the DINOv2 backbone parameters remain fixed during both training and inference. Future work will explore smaller backbone architectures or model compression techniques to further reduce the total inference footprint for edge deployment.

#### 3.1.2 Transformer-ODE branch for continuous temporal modeling

**Figure 3:**
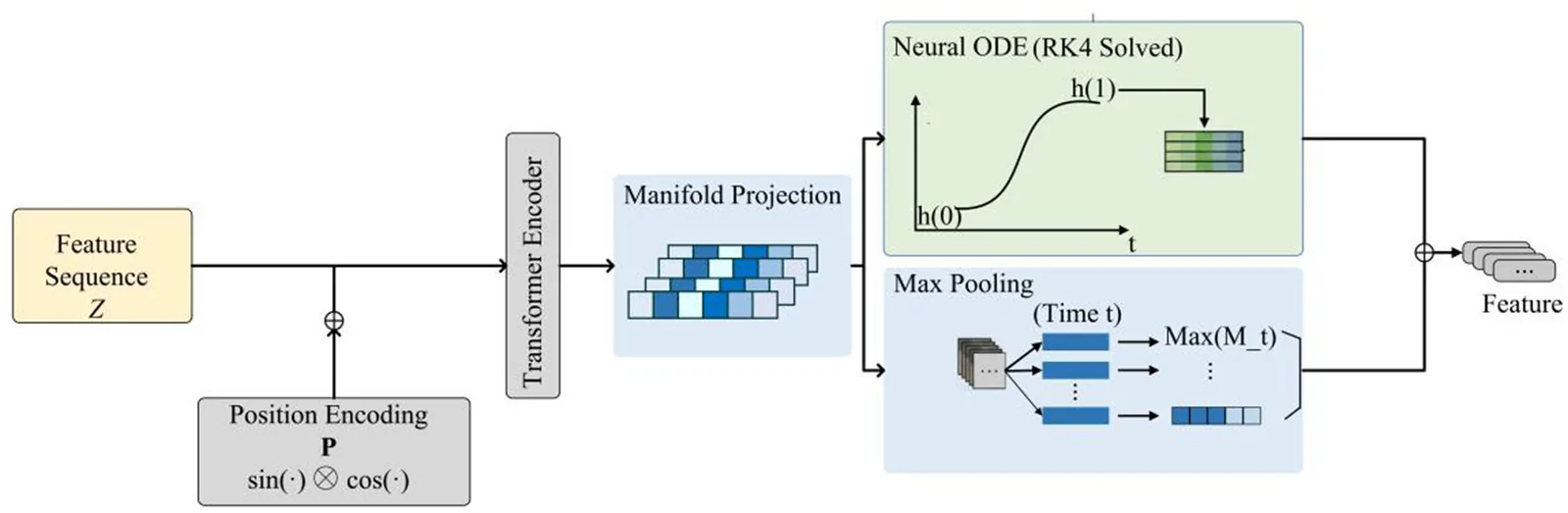
Detailed structure of the Transformer-ODE branch.


The Transformer-ODE branch maps discrete feature sequences into a continuous dynamical system defined by Neural ODE to compensate for temporal information gaps caused by discrete sampling. The input feature sequence *X*∈ℝ^*B*×*T*×*D*^ is projected into a low-dimensional continuous differentiable manifold via a lightweight Transformer encoder. The lightweight Transformer encoder consists of 1 layer with 4 heads and hidden dimension *d*_*h*_ = 64. This process generates the low-dimensional manifold feature sequence *M*. Global max pooling along the temporal dimension extracts the most discriminative instantaneous behavioral features, as shown in Figure 3. The temporal global max-pooling operation is expressed in Equation 3.

$$\begin{array}{c} \underset{t}{\max}\left(M\right)=\underset{t}{\max}M_{:,t,:} \end{array}$$ (3)

where $\underset{t}{\max}\left(M\right)\in {\mathbb{R}}^{B\times d_{h}}$ denotes the pooled feature vector, and *d*_*h*_ denotes the hidden dimension of the manifold space. The continuous evolution of the hidden state within the normalized time interval [0, 1] is described by an ordinary differential equation. The terminal state *h*(1) is solved via the fourth-order Runge-Kutta integrator. The instantaneous feature and continuous dynamic terminal state are fused using Equation 4.


$$\begin{array}{c} F_{to}=\underset{t}{\max}\left(M\right)+h\left(1\right) \end{array}$$ (4)

This fusion preserves smooth dynamic trends from Neural ODE and discriminative instantaneous information from Transformer encoding, enhancing temporal feature robustness.

#### 3.1.3 Fourier attention branch for global periodic feature modeling

The Fourier attention branch performs adaptive weighting on frequency-domain representations to capture global periodic patterns. For each channel of the temporal feature matrix *X*, the discrete Fourier transform is performed along the temporal dimension, converting time-domain sequences into complex frequency-domain coefficients *U*. The amplitude spectrum is calculated using Equation 5.


$$\begin{array}{c} S=|U| \end{array}$$ (5)

where *S*∈ℝ^*T*×*D*^ denotes the amplitude spectrum. Low-frequency bands correspond to static behaviors, middle-frequency bands to rhythmic motions, and middle-high frequency bands to diarrhea-related oscillations. The global frequency attention weights are generated using Equation 6.


$$\begin{array}{c} \alpha =\text{Softmax}\left(\phi \left({\text{Pool}}_{c}\left(S\right)\right)\right) \end{array}$$ (6)

where ϕ(·) denotes the frequency-weight mapping function, and Pool_*c*_(·) denotes channel-wise global average pooling. The amplitude spectrum is adaptively weighted using Equation 7.


$$\begin{array}{c} S^{g}=\alpha ⊙S \end{array}$$ (7)

where ⊙ denotes element-wise multiplication. A sub-band modulation mechanism refines frequency-band contributions, and the fused spectral features are projected to the target dimension to output *F*_*fa*_.

#### 3.1.4 Wavelet attention branch for local abrupt feature modeling

The Wavelet attention branch employs a multi-scale Haar wavelet transform to capture local transient abnormalities. A 3-level Haar wavelet decomposition generates low-frequency $f_{low}^{l}$ and high-frequency $f_{high}^{l}$ coefficients, *l* ∈ {0, 1, 2}, as shown in Equation 8.


$$\begin{array}{l} \left(f_{low}^{l},f_{high}^{l}\right)=TGAP_{t}\left(DWT\left(X^{l}\right)\right) \end{array}$$ (8)

where *DWT*(·) denotes discrete wavelet transform, and *TGAP*_*t*_(·) denotes temporal global average pooling. The pooled low-frequency and high-frequency statistical features construct the global description vector *f*_*global*_, as defined in Equation 9.


$$\begin{array}{l} f_{global}=\alpha f_{low}^{l}+\left(1-\alpha \right)f_{high}^{l} \end{array}$$ (9)

where α denotes a learnable coefficient that balances the contributions of the low-frequency and high-frequency features. Then, the global description vector *f*_*global*_ passes through two linear mappings and Softmax activation to generate the scale attention weights *w*, as described in Equation 10.


$$\begin{array}{l} w=Softmax\left(W_{2}\left(W_{1}f_{global}\right)\right) \end{array}$$ (10)

where *W*_1_ and *W*_2_ denote learnable weight matrices. Weighted wavelet coefficients are linearly transformed to output the branch feature *F*_*wa*_, as specified in Equation 11.


$$\begin{array}{l} F_{wa}=\left(w⊙DWT\left(X\right)\right)W_{wa}+b_{wa} \end{array}$$ (11)

where *W*_*wa*_ and *b*_*wa*_ denote learnable parameters, enabling precise capture of local abrupt features with minimal parameters.

#### 3.1.5 Multi-domain feature fusion and classification

The outputs *F*_*to*_, *F*_*fa*_, and *F*_*wa*_ of three branches are concatenated into a unified feature vector. The outputs of the three branches are concatenated using Equation 11.


$$\begin{array}{c} F_{cat}=\text{Concat}\left[F_{to},F_{fa},F_{wa}\right] \end{array}$$ (12)

The fused features are refined via layer normalization, linear transformation, GELU activation, and Dropout. They are then fed into a linear classifier to generate category logits for calf behavior classification. This low-dimensional fusion scheme maintains model lightweightness. Complementary integration of temporal, global frequency, and local time-frequency features is achieved at the same time.

### 3.2 Evaluation metrics

Four standard classification metrics are adopted to evaluate recognition performance. Accuracy (ACC) is the ratio of correctly predicted samples to total samples. Precision (P) is the proportion of true positive samples among predicted positive samples. Precision is calculated using Equation 12.


$$\begin{array}{c} P=\frac{TP}{TP+FP} \end{array}$$ (13)

where *TP* denotes true positives and *FP* denotes false positives. Recall (R) is the ratio of correctly identified positive samples to all actual positive samples. Recall is calculated using Equation 13.


$$\begin{array}{c} R=\frac{TP}{TP+FN} \end{array}$$ (14)

where *FN* denotes false negatives. The F1-score is the harmonic mean of precision and recall. The F1-score is calculated using Equation 14.


$$\begin{array}{c} \text{F1-score}=\frac{2\cdot P\cdot R}{P+R} \end{array}$$ (15)

The total number of parameters is used to measure model lightweightness, and normalized confusion matrices visualize classification performance across behavior categories.

### 3.3 Loss function

Focal Loss with label smoothing is employed as the classification loss to prioritize hard-to-classify samples and enhance generalization. The focal loss with label smoothing is formulated in Equation 15.


$$\begin{array}{c} L_{FL}=\frac{1}{B}\overset{B}{\underset{i=1}{\sum }}{\left(1-p_{t_{i}}\right)}^{\gamma }\cdot \text{CE}\left(x_{i},y_{i}\right) \end{array}$$ (16)

where *B* denotes the batch size, *p*_*t*_*i*__ denotes the predicted probability of the ground-truth class, γ = 2.0 denotes the focusing coefficient, and CE(*x*_*i*_, *y*_*i*_) denotes the cross-entropy loss with label smoothing. The cross-entropy loss with label smoothing is calculated using Equation 16.


$$\begin{array}{c} \text{CE}\left(x,y\right)=-\overset{K}{\underset{k=1}{\sum }}q_{k}\log p_{k} \end{array}$$ (17)

where $q_{k}=\left(1-\varepsilon \right)\delta_{y,k}+\frac{\varepsilon }{K}$. Here, ε = 0.1 denotes the smoothing factor, *K* denotes the number of categories, and *p*_*k*_ denotes the predicted probability of class *k* given the input *x*. Additionally, δ_*y, k*_ represents the Kronecker delta, which equals 1 if *y* = *k*, and 0 otherwise.

## 4 Experiment and analysis

### 4.1 Comparative experiments and performance analysis of different network models

In order to evaluate the performance of the proposed DTOFW model, this section conducts comparative experiments and analysis. ConvNeXt, TransNeXt, ViT, and PVT are selected as four mainstream backbone networks for comparison. All comparison results are presented in Tables 2, 3.

**Table 2:** Overall performance comparison of different models.

Model	Accuracy (%)	Loss	Precision (%)	Recall (%)	F1 score (%)	Parameters (M)
ConvNeXt	87.46	0.3104	88.46	86.65	87.30	55.66
TransNeXt	86.61	0.3199	88.86	85.07	85.89	54.34
ViT	87.80	0.2860	89.32	87.10	87.87	32.00
PVT	89.83	0.2830	90.89	89.56	89.99	39.82
DTOFW	95.32	0.1342	94.93	95.56	95.15	1.33

The Parameters (M) column reports trainable parameters. For DTOFW, this value refers only to the trainable tri-branch fusion head, while the frozen DINOv2 backbone is excluded from this column.

In the two-stage framework, the value of 1.33M represents the supervised trainable cost of the fusion head. During inference, the complete DTOFW system still relies on the frozen DINOv2 backbone for frame-level feature extraction. Therefore, the term lightweight mainly refers to the trainable parameters and training cost of the fusion head, rather than the total cost of the complete inference pipeline. This distinction avoids an unfair comparison between the trainable fusion-head cost of DTOFW and the complete parameter counts of the frame-based baseline models.

The data in Table 2 indicate that the proposed DTOFW model outperforms the tested baseline models across all performance metrics. Compared with the tested baseline models in this study, DTOFW achieves 95.32% recognition accuracy and improves accuracy by 7.86, 8.71, 7.52, and 5.49 percentage points over ConvNeXt, TransNeXt, ViT, and PVT, respectively. These results indicate the effectiveness of the proposed lightweight time-frequency fusion head under the current experimental setting. In addition, 5-fold cross-validation yields a mean accuracy of 94.8% with a standard deviation of 0.3%, indicating stable performance across different splits. Notably, the precision and recall for the diarrhea class remain consistently high across all folds, further demonstrating the model's reliability in identifying this minoritarian behavior without severe overfitting. Furthermore, the DTOFW model achieves a loss value of 0.1342, representing a reduction of 0.1488 to 0.1857 compared to the other four models. Regarding precision, recall, and F1 score, the DTOFW model attains 94.93%, 95.56%, and 95.15% respectively. It surpasses the comparison models by 4.04% to 6.47%, 6.00% to 10.30%, and 5.16% to 8.86%, respectively. This result demonstrates improved recognition accuracy and robustness in calf diarrhea behavior recognition. Moreover, DTOFW improves recognition accuracy while keeping the trainable fusion head at only 1.33 million parameters. ConvNeXt contains 55.66 million parameters and requires end-to-end training. In contrast, the compact fusion head reduces training computational cost.The full system still depends on a frozen DINOv2 backbone during inference. Therefore, DTOFW shows potential for deployment on resource-constrained devices. Further tests on real edge hardware remain necessary to evaluate full-system performance.

Table 3 demonstrates improved recognition performance of the DTOFW model in the calf multi-category behavior recognition task. Specifically, the model achieves the highest average precision, average recall and average F1-score, and obtains perfect precision, recall and F1-score in recognizing the two behaviors of diarrhea and lying. For the recognition of eating, standing and walking behaviors, although its results are slightly inferior to those of the former two, it still reaches an excellent and competitive level. Overall, these results indicate that DTOFW provides improved recognition performance for calf behavior classification and confirms the effectiveness of the proposed multi-domain feature fusion strategy.

**Table 3:** Category-wise recognition performance of different models.

Model	Metric	Category					Avg Prec	Avg Rec	Average F1
		Diarrhea-related	Eating	Lying	Standing	Walking			
ConvNeXt	Precision	93.02	92.68	94.67	75.95	85.96	88.46	85.99	89.19
	Recall	80.00	80.85	95.95	85.71	87.46			
	F1-Score	86.02	86.36	95.30	90.74	87.52			
TransNeXt	Precision	96.20	82.10	90.10	78.50	87.40	86.86	84.98	85.79
	Recall	92.50	75.30	91.20	86.30	79.60			
	F1-Score	94.30	78.50	90.65	82.20	83.30			
ViT	Precision	97.56	91.49	94.81	73.17	89.58	89.32	87.10	87.87
	Recall	80.00	91.49	98.65	85.71	79.63			
	F1-Score	87.91	91.49	96.69	78.95	84.31			
PVT	Precision	100	87.76	95.89	78.21	92.59	90.89	89.56	89.99
	Recall	82.00	91.49	94.59	87.14	92.59			
	F1-Score	90.11	89.58	95.24	82.43	92.59			
DTOFW	Precision	100	84.91	100	97.14	92.59	94.93	95.56	95.15
	Recall	100	95.74	100	89.47	92.59			
	F1-Score	100	90.00	100	93.15	92.59			

**Figure 4:**
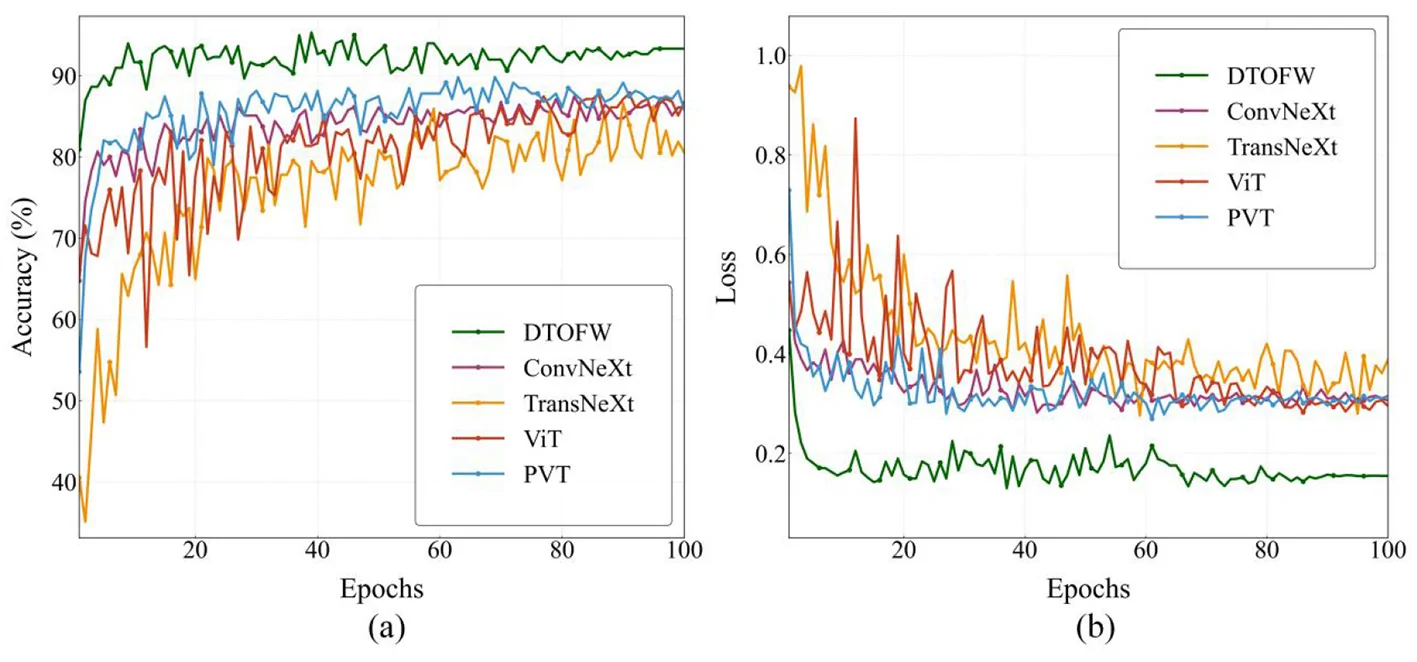
Accuracy and loss curves during training: **(a)** accuracy curves of DTOFW, ConvNeXt, TransNeXt, ViT, and PVT; **(b)** loss curves of DTOFW, ConvNeXt, TransNeXt, ViT, and PVT.

To illustrate the performance disparities between models more intuitively, this study visualized the training dynamics of different network architectures, as depicted in Figure 4. As depicted in Figure 4a, the accuracy curve of DTOFW consistently outperforms those of the other comparison models throughout the entire training cycle. It simultaneously exhibits faster ascent and superior convergence stability. Figure 4b indicates that its loss value rapidly decreases during the initial training phase, converging to an exceptionally low level of 0.1342. The entire optimization process remains smooth, with no rebound fluctuations observed.

**Figure 5:**
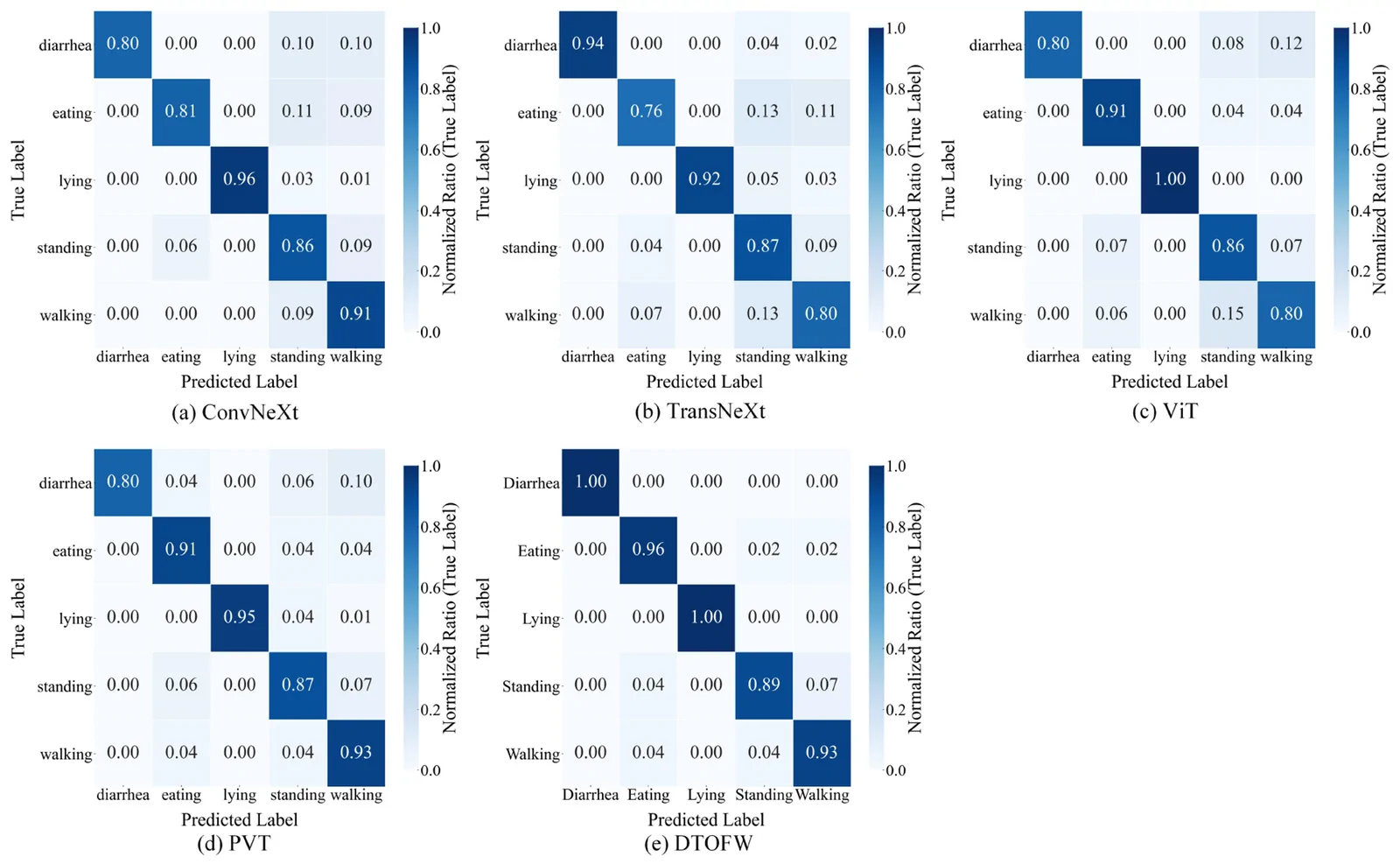
Normalized confusion matrices of different models: **(a)** ConvNeXt; **(b)** TransNeXt; **(c)** ViT; **(d)** PVT; and **(e)** DTOFW. The confusion matrices are row-normalized by true class.

To validate the performance of different network models in calf diarrhea behavior recognition, this study employs a normalized confusion matrix as a visualization tool. It clearly illustrates the relationship between each model's predicted behaviors and the actual behaviors. Figure 5 shows that the ConvNeXt, TransNeXt, ViT, and PVT models perform reasonably well on the lying behavior. Their accuracy in the core diarrhea recognition task ranges only from 0.80 to 0.94. In addition, they tended to misclassify eating behavior as standing or walking. In contrast, the DTOFW model demonstrates superior performance. It achieves perfect recognition of both diarrhea and lying, with an accuracy of 1.00, and also attains a high recognition rate of 0.96 for eating. The results indicate that the complementary design of time-frequency and global-local features enables the model to precisely capture subtle postural abnormalities and abrupt movement changes. Although the diarrhea category contains only 271 samples, DTOFW achieves perfect precision and recall for this class. This suggests that the time-frequency features extracted by the model are highly discriminative even under limited data conditions. To further assess robustness, we performed 5-fold cross-validation. The results are consistent, with a mean accuracy of 94.8% and a standard deviation of 0.3%, suggesting that the model is unlikely to overfit the small diarrhea subset.

Overall, the proposed DTOFW demonstrates more favorable recognition results in challenging calf behavior analysis. This advantage can be attributed to the joint effect of the continuous temporal modeling and dual-frequency attention mechanisms. First, the Transformer-ODE branch strengthens temporal feature representation by modeling continuous dynamic evolution, overcoming the information loss caused by discrete frame sampling. Second, the parallel frequency branches focus on different scales: the Fourier attention captures global periodic behavior patterns, whereas the Wavelet attention addresses local transient postural abnormalities. Third, their joint effect further improves the discriminative capacity for visually similar actions. Consequently, DTOFW preserves continuous behavioral trends and fine abnormal details more effectively, thereby achieving high accuracy on the Jinnan dataset with a compact trainable head, compared with the tested baselines.

### 4.2 Validating the effectiveness of DINOv2

To validate the feature extraction advantages of the DINOv2 pre-trained backbone in calf diarrhea behavior recognition, this section conducts comparative experiments. DINOv2 is fused with four mainstream networks, namely ConvNeXt, TransNeXt, ViT, and PVT. Four fusion models are constructed, namely DINOv2+ConvNeXt, DINOv2+TransNeXt, DINOv2+ViT, and DINOv2+PVT. Comparative experiments are conducted under identical conditions on these models and the original backbone models without fusion. The enhancement effect of DINOv2 on recognition performance is quantitatively analyzed. Experimental results are presented in Table 4.

**Table 4:** Performance comparison of models with and without frozen DINOv2 features.

Model	Accuracy (%)	Loss	Precision (%)	Recall (%)	F1 score (%)	Parameters (M)
ConvNeXt	87.46	0.3104	88.46	86.65	87.30	55.66
DINOv2+ConvNeXt	94.65	0.1790	95.08	94.29	94.63	7.91
TransNeXt	86.61	0.3199	88.73	85.26	86.29	54.10
DINOv2+TransNeXt	94.98	0.1415	94.91	94.60	94.74	11.04
ViT	87.80	0.2860	89.32	87.10	87.87	32.00
DINOv2+ViT	93.65	0.1942	93.56	93.90	93.61	2.82
PVT	89.83	0.2830	90.89	89.56	89.99	39.82
DINOv2+PVT	93.65	0.1948	93.93	93.07	93.43	11.44

The Parameters (M) column reports trainable parameters. For DINOv2-based models, the frozen DINOv2 backbone is excluded from this column.

Table 4 shows that integrating the pre-trained DINOv2 module consistently improves all four backbone networks across all evaluation metrics. Specifically, the accuracy gains range from 3.82 to 8.37 percentage points, while the loss decreases by 0.0882 to 0.1784. Precision, recall, and F1-score increase by 3.04–6.62, 3.51–9.34, and 3.44–8.45 percentage points, respectively. Among all models, DINOv2+TransNeXt achieves the best overall performance, with 94.98% accuracy, 0.1415 loss, 94.91% precision, 94.60% recall, and 94.74% F1-score. In addition, the fusion models require substantially fewer trainable parameters than their original counterparts, since the DINOv2 backbone is frozen during training and therefore excluded from the parameter count. Notably, DINOv2+ViT achieves competitive performance with only 2.82M trainable parameters, indicating a favorable trade-off between model efficiency and predictive performance.

**Figure 6:**
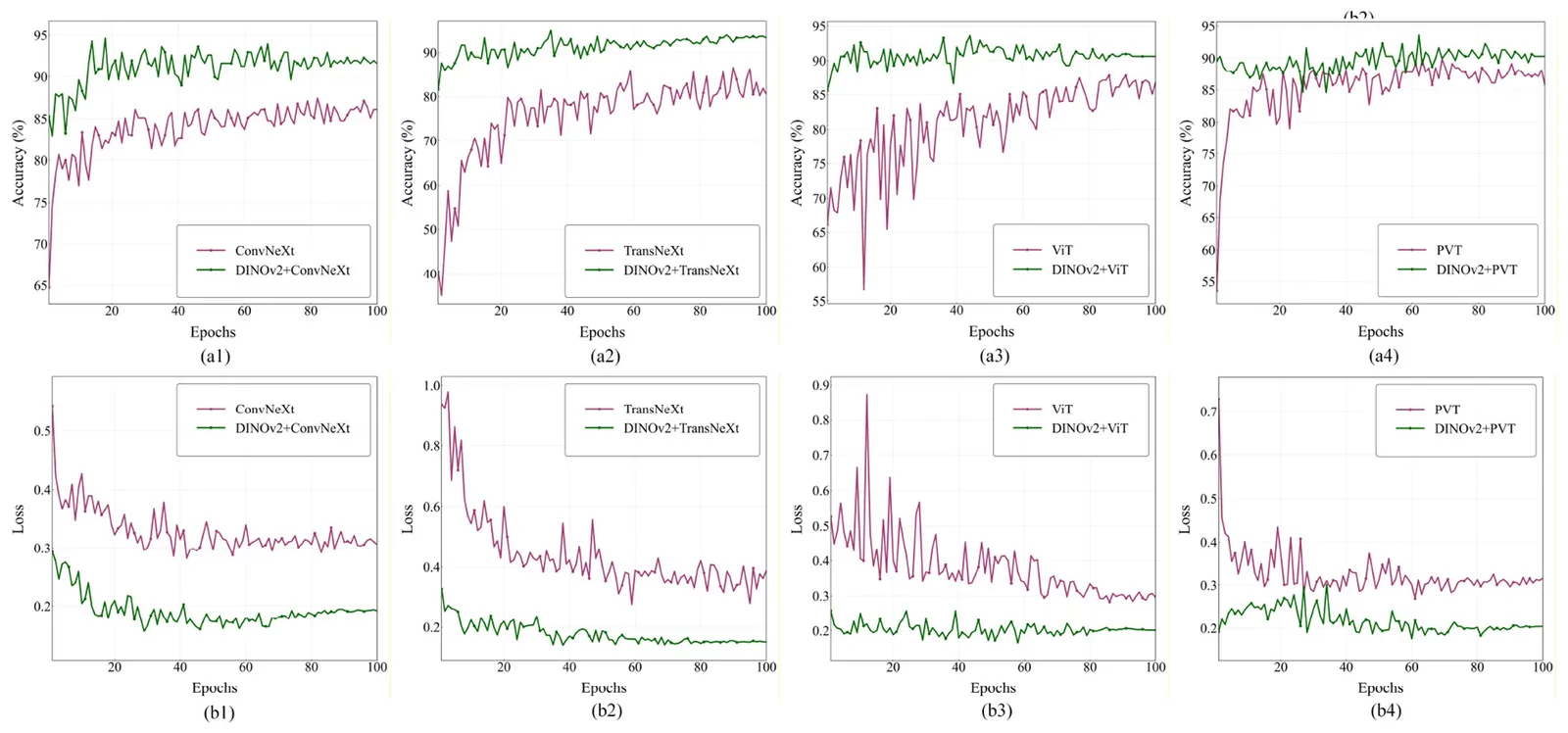
Comparison of recognition accuracy and loss between the baseline models and their DINOv2-enhanced versions: **(a1)** ConvNeXt, **(a2)** TransNeXt, **(a3)** ViT, and **(a4)** PVT accuracy curves; **(b1)** ConvNeXt, **(b2)** TransNeXt, **(b3)** ViT, and **(b4)** PVT loss curves.

To illustrate DINOv2's enabling effects more intuitively, the accuracy and loss value trends of models before and after integration are visualized (as shown in Figure 6). Figure 6a compares the accuracy of models before and after integrating DINOv2. It is evident that the accuracy curves of all integrated models significantly exceed those of the original models, rising more rapidly during training and converging to a more stable plateau. Figure 6b presents the loss comparison. The loss curve of the models after integrating DINOv2 declines rapidly from the early training stages and consistently remains within a low-level range. In contrast, the original models not only exhibit a high initial loss value but also demonstrate a markedly slower rate of decline.

These results fully validate the efficacy and superiority of DINOv2 in calf diarrhea behavior recognition tasks. On the one hand, DINOv2 uses self-supervised pre-trained features to accurately capture subtle visual characteristics of calf diarrhea behavior, including posture and movement rhythm. This overcomes the limitations of traditional backbone networks in extracting behavior-specific features in calf farming scenarios. On the other hand, its lightweight feature extraction approach challenges the idea that high performance must rely on a large number of parameters. It achieves improvements in both performance and efficiency.

In conclusion, the integration of the frozen DINOv2 backbone demonstrates more favorable feature extraction results under complex farm environments. This advantage can be attributed to the joint effect of robust self-supervised pre-training and strategic feature decoupling. First, DINOv2 strengthens frame-level semantic representation by providing highly discriminative visual features that are resistant to illumination and background interference. Second, freezing this backbone ensures that the original structural knowledge is preserved, while the downstream lightweight head explicitly addresses the time-frequency behavioral dynamics. Third, their joint effect further prevents the network from overfitting on limited sample sizes. As a result, this paradigm preserves high-level spatial semantics and stabilizes the training process more effectively, thereby improving both recognition generalization and computational efficiency.

### 4.3 Ablation studies and module effectiveness validation

In this section, a series of ablation experiments are conducted to systematically assess the effectiveness of the four core modules within the DTOFW network, namely Transformer, ODE, FA, and WA. Specifically, using only one module or an incomplete module combination has clear limitations. Under this setting, the model cannot effectively capture temporal continuity, continuous dynamic changes, global frequency patterns, and local transient features at the same time. Consequently, the performance of the model declines markedly in the task of calf diarrhea behavior recognition. Therefore, the complete DTOFW model with all four modules is used as the baseline. Two sets of ablation experiments are then conducted. These experiments further validate the independent contributions and synergistic effects of each module.

#### 4.3.1 Single-module retention ablation experiments

In this subsection, a series of single-module removal ablation experiments are conducted to validate the independent contribution of each core module in DTOFW. In these experiments, only one module is retained each time, while the other modules are removed. The necessary preprocessing operations are still preserved to ensure that the retained module can function properly. The complete DTOFW model with all four modules is used as the reference model. The experimental results are shown in Table 5.

**Table 5:** Ablation study results of different module combinations.

Index	Block				Accuracy(%)	Loss	Precision(%)	Recall(%)	F1 Score(%)
	Transformer	ODE	FA	WA					
1	√	×	×	×	91.30	0.2051	90.66	91.02	90.68
2	×	√	×	×	93.31	0.1574	92.90	92.95	92.91
3	×	×	√	×	94.31	0.1892	95.13	93.43	94.06
4	×	×	×	√	94.31	0.1599	94.42	93.93	94.16
5	√	√	√	√	95.32	0.1342	94.93	95.56	95.15

Based on Table 5, when only one core module is retained, the performance of the model decreases to different degrees. When only the Transformer module is used, the model shows the weakest performance. The accuracy is 91.30%, and the loss reaches 0.2051. This result indicates that discrete temporal modeling alone is not sufficient for calf diarrhea behavior recognition. It cannot fully describe the complex temporal changes in behavior sequences.

When only the ODE module is retained, the model achieves better performance than the Transformer-only setting. The accuracy rises to 93.31%, and the loss decreases to 0.1574. This result shows that continuous dynamic modeling can better capture the evolution process of behavior features. It also helps compensate for the temporal information gaps caused by discrete sampling.

When only the FA module is retained, the model reaches an accuracy of 94.31%. This result suggests that frequency-domain modeling is effective for behavior recognition. The FA module can capture global periodic patterns in the feature sequence. However, its loss is 0.1892, which is still relatively high. This indicates that global frequency information alone is not enough to ensure the best recognition performance.

When only the WA module is retained, the model also achieves an accuracy of 94.31%. At the same time, its loss further decreases to 0.1599. Compared with FA, WA shows a stronger ability to capture discriminative features. This is mainly because the WA module is more effective in describing local transient changes and subtle abnormal patterns in calf diarrhea behavior.

When all four modules are jointly introduced, the model obtains the best overall performance. The accuracy reaches 95.32%, the F1-score reaches 95.15%, and the loss decreases to 0.1342. These results demonstrate that each module has its own unique role in feature extraction. They also confirm that the four modules are complementary rather than redundant. Therefore, the full DTOFW framework provides the most effective feature representation for calf diarrhea behavior recognition.

#### 4.3.2 Stepwise module overlay ablation experiments

This section quantitatively analyzes the synergistic gains from multi-module integration through progressive core module stacking, with results presented in Table 6.

**Table 6:** Incremental performance comparison of module combinations and the final model.

Model	Accuracy (%)	Loss	Precision (%)	Recall (%)	F1 score (%)
Transformer	91.30	0.2051	90.66	91.02	90.68
Transformer + ODE	93.65	0.1727	93.62	92.85	92.91
Transformer + ODE + FA	93.98	0.1650	93.77	93.72	93.61
Transformer + ODE + WA	94.31	0.1380	94.45	94.39	94.40
Transformer + FA +WA	94.31	0.1623	94.42	94.06	94.22
DTOFW	95.32	0.1342	94.93	95.56	95.15

In this subsection, stepwise module aggregation ablation experiments are conducted to further evaluate the synergistic effects of multi-module integration in DTOFW. Starting from the Transformer module, additional modules are introduced progressively to examine their influence on model performance. The experimental results are shown in Table 6.

As shown in Table 6, the Transformer-only model achieves an accuracy of 91.30% with a loss of 0.2051. This result indicates that the Transformer can provide basic temporal modeling ability, but its representation capacity remains limited when used alone. After the ODE module is added, the accuracy increases to 93.65% and the loss decreases to 0.1727. This improvement suggests that continuous dynamic modeling can effectively complement discrete temporal modeling and enhance the description of behavioral evolution.

Further introduction of frequency-domain modules leads to additional performance gains. With the FA module, the model reaches 93.98% accuracy and a loss of 0.1650, indicating that FA contributes useful global frequency information. By contrast, the Transformer+ODE+WA configuration achieves 94.31% accuracy with a lower loss of 0.1380. This result shows that WA is more effective than FA in the current task, mainly because it is better at capturing local transient changes and subtle abnormal patterns in calf diarrhea behavior.

After all four modules are integrated, the complete DTOFW model obtains the best overall performance. The accuracy reaches 95.32%, and the loss decreases to 0.1342. Meanwhile, precision, recall, and F1-score also achieve their optimal values. These results demonstrate that model performance improves progressively as the modules are aggregated, and they further confirm the complementary roles of Transformer, ODE, FA, and WA in multi-domain feature extraction.

Overall, the complete DTOFW framework demonstrates more favorable multi-domain feature fusion results compared to any partial module combination. This advantage can be attributed to the joint effect of the Transformer-ODE, Fourier Attention (FA), and Wavelet Attention (WA) modules. First, the Transformer-ODE strengthens temporal continuity by mapping discrete sequences into a continuous dynamic space. Second, within the time-frequency enhancement mechanism, the FA branch focuses on global periodic rhythms, whereas the WA branch addresses local abrupt changes and non-stationary signals. Third, their joint effect further improves information orthogonality and comprehensive behavioral representation without requiring high-dimensional concatenation. Therefore, DTOFW preserves global dynamic trends and local fine-grained features more effectively, thereby maximizing the classification performance for subtle diarrhea-related behaviors.

## 5 Discussion

This paper proposes DTOFW, a lightweight three-branch spatiotemporal fusion network for calf diarrhea recognition. Addressing two key challenges, the framework targets temporal continuity loss caused by discrete sampling and the joint modeling of periodic and sudden behaviors. Experimental results on the custom Jinnan calf dataset show that DTOFW achieves 95.32% accuracy with 1.33M trainable parameters in the tri-branch fusion head. Compared with the tested baseline models, DTOFW improves accuracy by 5.52%–8.71% and reduces loss values by more than 53%. The improved performance mainly arises from the synergy of three complementary branches. Together, these branches characterize diarrhea behaviors across continuous time, global frequency, and local spatiotemporal domains.

At the temporal level, the Transformer-ODE branch overcomes the limitations of traditional discrete-time modeling. By embedding continuous temporal dynamics into feature learning, it restores missing information between video frames. Continuous differential equations further enable the network to capture the true evolution of behaviors. This capability is particularly well suited to the slow and steady-state changes observed during diarrhea. In parallel, the Fourier Attention branch enables global perception of periodic behaviors in the frequency domain. Rhythmic movements caused by abdominal discomfort usually require long temporal sequences for effective modeling in the time domain. Frequency-domain attention compresses these patterns into key frequency bands to capture global dependencies more efficiently. For transient behavioral features, the Wavelet Attention branch performs multi-scale localization. Targeting non-stationary events such as sudden diarrhea or walking, this approach employs adaptive wavelet coefficients. These coefficients balance the time and frequency domains and compensate for the limitations of Fourier transforms in handling non-stationary signals. Within a low-dimensional space, the three branches integrate complementary features without introducing the computational burden associated with high-dimensional concatenation. Information orthogonality is preserved simultaneously. Owing to these synergies, DTOFW achieves superior performance while maintaining a highly compact architecture.

Compared with existing methods, DTOFW exhibits clear advantages in both accuracy and efficiency. Recent CNN-LSTM-based approaches for calf behavior recognition typically achieve 86%–90% accuracy with 2–5M parameters, whereas DTOFW exceeds 95% accuracy with fewer parameters. Unlike most current methods that rely on discrete frame sampling and temporal pooling, the proposed framework captures continuous behavioral evolution more effectively. ODE-based modeling therefore aligns more closely with real biological behavior processes. In addition, only a limited number of existing studies combine Fourier and Wavelet transform attention mechanisms. By demonstrating the complementary value of multi-scale frequency-domain features, DTOFW offers a new technical perspective for fine-grained animal behavior recognition.

Technology-based behavior recognition cannot replace good husbandry, disease prevention, routine inspection, or appropriate animal care. The proposed DTOFW model should be regarded as a preliminary video-based behavior recognition method. Its practical value depends on whether the recognition results can be combined with farm management and timely human intervention. Despite its excellent performance on the current dataset, further validation of the generalization and robustness of DTOFW remains necessary. Data used in this study were collected from a single farming scenario with fixed camera angles and relatively stable lighting conditions. Real-world environments, however, often involve occlusion, group interference, abrupt illumination changes, and camera vibration, all of which have not yet been systematically evaluated. Large-scale and representative cattle behavior datasets covering different breeds, ages, and climatic conditions are therefore needed to support a more comprehensive assessment of model generalization. Another limitation lies in the exclusive use of RGB video signals, which may lead to performance degradation under nighttime or occluded conditions. To address this issue, future work will incorporate multimodal information, including wearable accelerometer signals, environmental sensing data, and infrared thermal imaging. Cross-modal knowledge distillation may further enhance robustness while preserving the lightweight structure of the model. Distribution shifts across farms, caused by differences in ground textures, fence structures, and camera viewpoints, also remain an important challenge. Exploration of transfer learning strategies based on domain adaptation or domain generalization will be essential for reducing deployment costs in new environments. Overall, DTOFW provides an efficient and lightweight paradigm for fine-grained cattle behavior recognition. Practical ranch deployment will require continued advances in data scale, modality diversity, and transferability.

A limitation of this study is that the current dataset was annotated at the video-clip level and did not include complete veterinary clinical examination records, physiological measurements, fecal scoring, individual-level treatment records, or environmental measurements. Therefore, the proposed method should be regarded as a visual behavior-recognition approach for auxiliary health monitoring, rather than a diagnostic tool for confirming calf diarrhea. Future work will incorporate individual identity tracking, veterinary diagnosis, fecal scoring, treatment information, and environmental factors to support more rigorous clinical interpretation.

## 6 Conclusions

This study presents DTOFW, a lightweight tri-branch time-frequency fusion network for accurate and efficient calf diarrhea behavior recognition. The network leverages frozen DINOv2 for high-level semantic feature extraction. Transformer-ODE, Fourier attention, and Wavelet attention branches are integrated to model continuous temporal dynamics, global periodic patterns, and local transient abnormalities simultaneously. Experimental results on the Jinnan calf dataset demonstrate that DTOFW achieves 95.32% accuracy with 1.33M trainable parameters in the tri-branch fusion head. The results indicate that DTOFW improves recognition performance over the tested baseline models while maintaining a compact trainable fusion head. Further evaluation on real edge hardware will be conducted in future work. The proposed model provides a feasible solution for fine-grained abnormal behavior recognition and may support intelligent health monitoring in smart livestock farming.

## Data Availability

The raw video data supporting the findings of this study are not publicly available due to farm confidentiality and data-use authorization restrictions. The data may be made available by the corresponding author upon reasonable request and with permission from the data provider.
