# Correction: DTOFW: a lightweight tri-branch time-frequency fusion network for video-based recognition of diarrhea-related calf behavior

**DOI:** 10.3389/fvets.2026.1961019

**Published:** 2026-09-03

**Authors:** Wangli Hao, Qingqing Li, Lingling Li, Yakui Xue, Meng Han, Yanhong Liu, FuZhong Li

**Affiliations:** 1 Faculty of Software Technologies, Shanxi Agricultural University, Jinzhong, Shanxi, China; 2 School of Information Science and Engineering, Shanxi Agricultural University, Jinzhong, Shanxi, China

**Keywords:** calf diarrhea recognition, DINOv2, lightweight network, neural ODE, time-frequency fusion

An error was identified in Section 3.1.4, “Wavelet attention branch for local abrupt feature modeling.” The methodological description and mathematical notation in (8)–(11) did not fully and accurately describe the implemented Wavelet attention branch. This correction is limited to the methodological description and mathematical notation and does not affect the implemented model architecture, dataset, experimental settings, numerical results, discussion, or conclusions of the published article.

A correction has been made to Section 3.1.4, “Wavelet attention branch for local abrupt feature modeling.” The entire subsection, including (8)–(11), has been replaced as follows:

## 3.1.4 Wavelet attention branch for local abrupt feature modeling

The Wavelet attention branch employs a multi-scale Haar wavelet transform to capture local transient abnormalities. A 3-level Haar wavelet decomposition generates low-frequency $f_{low}^{l}$ and high-frequency $f_{high}^{l}$ coefficients, *l* ∈ {0, 1, 2}, as shown in Equation 8.


$$\begin{array}{l} \left(f_{low}^{l},f_{high}^{l}\right)=TGAP_{t}\left(DWT\left(X^{l}\right)\right) \end{array}$$ (1)

where *DWT*(·) denotes discrete wavelet transform, and *TGAP*_*t*_(·) denotes temporal global average pooling. The pooled low-frequency and high-frequency statistical features construct the global description vector *f*_*global*_, as defined in Equation 9.


$$\begin{array}{l} f_{global}=\alpha f_{low}^{l}+\left(1-\alpha \right)f_{high}^{l} \end{array}$$ (2)

where α denotes a learnable coefficient that balances the contributions of the low-frequency and high-frequency features. Then, the global description vector *f*_*global*_ passes through two linear mappings and Softmax activation to generate the scale attention weights *w*, as described in Equation 10.


$$\begin{array}{l} w=Softmax\left(W_{2}\left(W_{1}f_{global}\right)\right) \end{array}$$ (3)

where *W*_1_ and *W*_2_ denote learnable weight matrices. Weighted wavelet coefficients are linearly transformed to output the branch feature *F*_*wa*_, as specified in Equation 11.


$$\begin{array}{l} F_{wa}=\left(w⊙DWT\left(X\right)\right)W_{wa}+b_{wa} \end{array}$$ (4)

where *W*_*wa*_ and *b*_*wa*_ denote learnable parameters, enabling precise capture of local abrupt features with minimal parameters.

The original version of this article has been updated.

